# Smart Handover: MHCAS’s Digital Clinical Handover Tool

**DOI:** 10.1192/bjo.2026.11179

**Published:** 2026-06-30

**Authors:** Torsten Wrigley, Mehtab Rahman

**Affiliations:** Central and North West London Mental Health Trust, London, United Kingdom

## Abstract

**Aims:**

The Mental Health Crisis Assessment Centre (MHCAS) at St Charles Hospital is a newly established acute psychiatric service within CNWL NHS Foundation Trust. Initial clinical and operational processes relied on parallel documents across multiple applications, highlighting the need for a safer and more efficient solution in a high-pressure clinical environment.

Aim 1 – Achieving Safe Digital Implementation: Successfully design and implement a safe, standardised digital handover tool using existing NHS IT infrastructure.

Aim 2 – Achieving Efficiency & Productivity: Demonstrate measurable improvements in clinical efficiency, workflow productivity, and multidisciplinary communication.

Aim 3 – Achieving Sustainability & Governance: Embed the digital handover tool into clinical governance processes to achieve sustainable “business as usual” practice.

**Methods:**

The MHCAS Digital Handover Tool was developed using three applications within the NHS Microsoft 365 infrastructure: SharePoint Lists, Power Automate, and Power BI. Share Point Lists replaced three legacy handover documents with a single ‘master’ patient list serving as the central operational record. Power Automate automated routine administrative tasks. Power BI dashboards drew live data from the patient list to provide real-time visibility of operational activity, patient flow, and internal performance indicators, creating a unified digital handover and monitoring system.

Evaluation focused on system adoption, data quality, efficiency, and operational performance. Quantitative measures included weekly patient entries, number of active users, documentation update frequency, estimated administrative time savings, and referral-to-arrival times. These measures were continuously captured and visualised through live Power BI dashboards throughout the first quality improvement cycle. Qualitative feedback on staff experience, communication, and perceived value was collected at cycle completion.

**Results:**

Six-week outcomes (15/12/2025–25/01/2026) included:

• Weekly patient entries increased from 15 to 61.

• 27 active multidisciplinary users.

• Documentation updates increased from 10.5 to 12.6 per patient per day.

• Data completeness >98%.

• All legacy handover documents retired within one week.

• 30% reduction in administrative workload.

• Median referral-to-arrival time reduced from 15 to 6 hours (60% reduction) despite increased referrals.

• 100% (9/9) staff reported improved communication and reduced administrative burden.

**Conclusion:**

The MHCAS Digital Handover Tool demonstrates that meaningful digital transformation in acute mental health services can be safely achieved using existing NHS infrastructure and governance frameworks. The intervention delivered measurable efficiency gains, improved operational oversight, enhanced clinical safety, and improved staff experience. Live operational data supports continuous quality improvement, while early stakeholder engagement facilitated rapid adoption. The approach is scalable and replicable, with discussions underway regarding application across inpatient, crisis, and liaison services. Beyond providing a more efficient and modern alternative to the current handover tool, the project highlights the largely untapped potential of automation already available and applicable to NHS systems.

